# Implementing Smartphone-Based Telemedicine for Cervical Cancer Screening in Uganda: Qualitative Study of Stakeholders’ Perceptions

**DOI:** 10.2196/45132

**Published:** 2023-10-02

**Authors:** Johnblack K Kabukye, Jane Namugga, Collins Jackson Mpamani, Andrew Katumba, Joyce Nakatumba-Nabende, Hanifa Nabuuma, Stephen Senkomago Musoke, Esther Nankya, Edna Soomre, Carolyn Nakisige, Jackson Orem

**Affiliations:** 1 SPIDER - The Swedish Program for ICT in Developing Regions Department of Computer and Systems Sciences Stockholm University Stockholm Sweden; 2 Uganda Cancer Institute Kampala Uganda; 3 Mulago Specialised Women and Neonatal Hospital Kampala Uganda; 4 Department of Electrical Engineering Makerere University Kampala Uganda; 5 Global Programs for Research and Training University of California San Francisco Kampala Uganda

**Keywords:** telemedicine, cervical cancer, screening, visual inspection with acetic acid, cervicography, Uganda, digital health

## Abstract

**Background:**

In Uganda, cervical cancer (CaCx) is the commonest cancer, accounting for 35.7% of all cancer cases in women. The rates of human papillomavirus vaccination and CaCx screening remain low. Digital health tools and interventions have the potential to improve different aspects of CaCx screening and control in Uganda.

**Objective:**

This study aimed to describe stakeholders’ perceptions of the telemedicine system we developed to improve CaCx screening in Uganda.

**Methods:**

We developed and implemented a smartphone-based telemedicine system for capturing and sharing cervical images and other clinical data, as well as an artificial intelligence model for automatic analysis of images. We conducted focus group discussions with health workers at the screening clinics (n=27) and women undergoing screening (n=15) to explore their perceptions of the system. The focus group discussions were supplemented with field observations and an evaluation survey of the health workers on system usability and the overall project.

**Results:**

In general, both patients and health workers had positive opinions about the system. Highlighted benefits included better cervical visualization, the ability to obtain a second opinion, improved communication between nurses and patients (to explain screening findings), improved clinical data management, performance monitoring and feedback, and modernization of screening service. However, there were also some negative perceptions. For example, some health workers felt the system is time-consuming, especially when it had just been introduced, while some patients were apprehensive about cervical image capture and sharing. Finally, commonplace challenges in digital health (eg, lack of interoperability and problems with sustainability) and challenges in cancer screening in general (eg, arduous referrals, inadequate monitoring and quality control) also resurfaced.

**Conclusions:**

This study demonstrates the feasibility and value of digital health tools in CaCx screening in Uganda, particularly with regard to improving patient experience and the quality of screening services. It also provides examples of potential limitations that must be addressed for successful implementation.

## Introduction

Cervical cancer (CaCx) remains one of the most common and most deadly cancers in low- and middle-income countries (LMICs), particularly in sub-Saharan Africa [1]. There were over 600,000 new CaCx cases worldwide in 2020, and 88% of those were in LMICs [2]. Similarly, there were 342,000 deaths due to CaCx worldwide in 2020, and 91% of those deaths occurred in LMICs, with sub-Saharan Africa being the only region with an increasing age-standardized death rate [1]. In Uganda, CaCx is the leading cancer, accounting for 35.7% of all cancer cases in females and 20.5% of all cancer cases overall [3].

CaCx is highly preventable through vaccination against human papillomavirus (HPV) and early screening. However, due to financial, sociocultural, and health system challenges, access to vaccines is limited and uptake is low in LMICs [4-7]. Organized screening programs [8] are also lacking in many LMICs, and therefore, screening is erratic and opportunistic [9-13]. The result is that the majority of CaCx cases in LMICs are diagnosed in advanced stages, when curative treatment is no longer possible or difficult to achieve [14-18]. For example, the rate of diagnosis at an advanced stage has been reported as 89.3% in Nigeria [19], 88% in Morocco [20], 80.9% in Nepal [20], 80.5% in Kenya [21], and over 80% in Malawi [20]. Similarly, high rates of late diagnosis have also been reported in Rwanda [22], Ethiopia [23], and Haiti [24].

In Uganda, data from a nationally representative sample of 6000 girls found that only 22% had been vaccinated against HPV [25], while studies from different regions of the country, particularly in rural areas, found HPV vaccination completion to be as low as 12% [26]. A recent study in urban Kampala, where there is better access to health services, higher mobilization and outreach services, and higher socioeconomic status, showed that 43% of the 201 girls surveyed had completed HPV vaccination [27]. Similarly, low CaCx screening rates of <5% have been reported among women in rural Uganda [28] and about 33%-44% in some high-risk groups, such as HIV-positive women [29,30].

Harnessing digital health tools and interventions has the potential to improve different aspects of CaCx screening and control in Uganda and other LMICs. For example, a common CaCx screening technique used in LMICs is visual inspection with acetic acid (VIA) [31]. However, VIA is limited by its subjective nature, and the accuracy of diagnosis varies depending on the experience and skill of the health worker [31,32]. Continuous supervision and quality assurance are also often needed after the initial training of health workers on how to perform VIA [33-36]. Consequently, several researchers have investigated different digital health interventions to improve the *accuracy of VIA*, for example, using smartphone-based colposcopy and cervicography to enable inexperienced screening staff to capture cervical images and share them with an expert for a second opinion [34-45]. Other studies have used cervical images to *train artificial intelligence* (AI) models, which could potentially substitute the expert when one is unavailable for a second opinion or supplement them [38,41,46]. In addition, similar telemedicine solutions have been used to *improve pathology services*, which is another major challenge not only for CaCx but also for cancer control in general [22,47,48]. Similar to cervicography, in telepathology, Pap smears or microscopy slides are scanned and shared with an expert for a second opinion or AI algorithms are used to assess the scanned slides to detect abnormalities. These telemedicine approaches are especially relevant in Uganda and similar LMICs because they allow cancer services to be brought closer to the population, especially in rural areas. In these countries, experts, such as gynecologists and oncologists, are few and limited to urban settings, yet the majority of the population lives in rural areas and is isolated by geographical terrains (mountains or water bodies), poor transport networks, or even civil unrest. Another potential key role of digital health in CaCx control is *enhancing clinical documentation and data management* [34,41,49]. Accurate and timely data are crucial for monitoring of screening programs, patient follow-up and tracking, and clinical research. Yet, adoption of electronic medical records (EMRs) in cancer care is low, and a lack of clinical data is often cited as a major challenge in cancer control in Africa [50].

The potential for digital health in Uganda and Africa at large is supported by the rapid penetration and adoption of digital innovations, such as smartphones, computers, and the internet, in this region. There is also a growing body of evidence on digital health solutions in Africa, particularly mobile health (mHealth), showing a positive impact on treatment adherence by patients, the provision of health education and awareness of the general public, data collection and reporting, drug supply chain and stock management, and disease surveillance [51-53]. However, most of the implementations have been isolated pilots, focused on infectious diseases, and have lacked robust evaluation methods. Most evaluations have focused on feasibility, usability, and acceptability, with limited focus and evidence on clinical outcomes. Moreover, despite several studies from other African countries [34-36,39,40,42-45,49,54,55], implementations of digital health in CaCx screening and diagnosis in Uganda are limited [48].

We implemented a smartphone-based store-and-forward telemedicine system to support CaCx screening in Uganda. It consisted of the Gynocular (Gynius Plus AB) [56] portable colposcopy unit coupled with the Samsung Galaxy J5 Pro with a 13-megapixel camera (Multimedia Appendix 1). We installed the Open Data Kit (ODK) [57] app on the smartphone for collecting clinical details, cervical images, and the nurse’s VIA result. The data were securely sent via mobile broadband to a centralized ODK Aggregate server (Multimedia Appendix 2) at the Uganda Cancer Institute (UCI) in Kampala. The main objective was to allow the screening nurses at rural clinics to consult a gynecologist in Kampala, as needed. At the start of this study, nurses were provided with refresher training on VIA and on the smartphone-based telemedicine system, and continuous supervision and mentorship were provided both remotely and by physical visits by the authors, who are gynecologists and digital health experts. Later, we used the collected cervical images to train an AI model, which was embedded in a custom-built Android app. This app replaced the ODK for data collection, as well as providing automated analysis of the cervical images by the AI model (Multimedia Appendix 3). This was in passive mode, whereby the diagnosis from the AI model was only provided after the nurses had input their diagnosis. Nurses were informed that this was a pilot and were encouraged to rely on their diagnosis or consult the gynecologist rather than basing care decisions on AI results. We also developed a new dashboard with the AI results (Multimedia Appendix 4).

The objective of this paper was to describe the key stakeholders' (screening clinic staff and patients) perceptions of the telemedicine system for CaCx screening. Understanding their perceptions is crucial for ensuring that the intervention addresses their needs and is aligned with their constraints and workflows [58-62]. Such stakeholder engagement is also crucial for stakeholder buy-in and ownership and for sustained adoption of the intervention [58-60,62-64]. We specifically explored usability issues as well as barriers to and facilitators of adoption. Our experience can help others who are planning similar projects on their choices of digital health tools and implementation strategies.

## Methods

### Setting and Locations

This study took place from 2019 to 2021 at the UCI in Mulago, Kampala, Uganda, and at its satellite clinics in Arua (West Nile region of Uganda, 8 hours by road from Kampala), Jinja and Mayuge (Eastern Uganda, 3 and 4 hours from Kampala, respectively), and Mbarara (Southwestern Uganda, 5 hours from Kampala). The UCI is a large national referral comprehensive cancer center; Arua, Jinja, and Mbarara are general referral hospitals; and Mayuge is a community cancer prevention and screening clinic. Before the study, CaCx screening clinics were operational at the UCI in Kampala, Mbarara, and Mayuge; therefore, the nurses here were more experienced in CaCx screening. Screening clinics at Jinja and Arua were operationalized during this study, and the nurses for these sites were trained at the UCI on VIA, cryotherapy, thermocoagulation, and related aspects of CaCx screening before being deployed at the respective satellite sites. Therefore, these nurses had limited experience with VIA.

We screened a total of 2682 women from the 5 sites over the course of the project, with 322 (12%) diagnosed as VIA positive by the frontline nurses. Images from 2174 (81.1%) women were reassessed by a gynecologist and were used in AI model training. The gynecologist determined that 313 (14.4%) women screened positive (VIA positive or suspicious for cancer), and the best overall AI model performance was at 98% sensitivity, 82% specificity, 90% accuracy, and 0.97 area under the receiver operator characteristic curve.

### Participants

Participants included patients who presented for CaCx screening (ie, adults >18 years old) and screening clinic staff (nurses who performed the screening and administrative staff). These individuals were selected because they are the most important stakeholders for the smartphone-based telemedicine intervention [58-60]. As this was a qualitative study, no statistical sample size calculation was performed. Rather, pragmatic considerations and saturation [65-69] informed the number of focus group discussion (FGD) sessions and participants per session. All the health workers at the screening clinics were involved since they alternate in providing care at the clinic. This also resulted in more health workers than patients in the participant population. However, since health workers are the main users of the system and were distributed across different clinics, this number was justifiable. Patients are mostly affected in a passive manner, that is, they do not have to learn to use the smartphone-based telemedicine system, and to many (especially those who have never gone through screening), screening with the telemedicine system would be the “normal” process. Therefore, we anticipated patients would not have many opinions, and saturation [65,66,69] was quickly reached. On the contrary, health workers had been used to routine screening without the telemedicine system and had to learn a new way of working. Nevertheless, patients are important stakeholders in mHealth interventions, and their values, preferences, and opinions need to be considered to ensure acceptance of the intervention [58-60,62].

### Data Collection and Analysis

The study was informed by the Consolidated Framework for Implementation Research (CFIR) [69-72], which provides a comprehensive theoretical framework for planning, implementation, and evaluation of interventions, such as digital health technologies. The CFIR covers constructs or issues, such as user characteristics or preferences, characteristics of the technology (how complex it is and how well it fits into the workflows or user characteristics), organizational context (including cultural beliefs and patient-provider interactions), and the implementation process (eg, training, supervision, and support), all of which can be facilitators or barriers. The FGDs therefore explored these issues and were semistructured to allow flexibility and free flow of ideas and for any new issues to emerge. The FGD guide is attached in Multimedia Appendix 5.

The FGDs were audio-recorded and transcribed by the first and second authors for analysis. A rapid deductive approach was followed [73,74] in which the transcripts (and field notes) were summarized, jointly discussed, and mapped to the CFIR constructs by the first 3 coauthors (JKK, JN, and CJM). We particularly paid attention to the perceived benefits or relative advantages of the smartphone-based telemedicine system, as well as concerns, such as privacy and confidentiality, and the fear of AI.

We supplemented the FGDs with the field observations of authors who were involved in the operation of the project, with insights from the clinical data collected during screening, and with an evaluation survey of the health workers at the end of the project. The evaluation survey assessed the system usability using the System Usability Scale (SUS) [75], as well as health workers’ rating of the value of the intervention.

### Ethical Considerations

The study was reviewed and approved by the UCI research ethics committee (UCIREC#17-2018) and was registered by the Uganda National Council for Science and Technology (UNCST; #HS400ES). All participants (patients and nurses) provided informed consent before participating in the study. The nurses also received training on good clinical practice and protection of human subjects. To ensure data privacy and confidentiality, the smartphones used in this study were password-protected, were strictly used for study purposes, had all other apps removed, and were always kept securely in the clinics. In addition, study identifiers were used instead of patient names in the ODK data collection forms. Participants received Ugandan Shilling (UGX) 20,000 (approximately US $5) as reimbursement for their time as per the UNCST guidelines.

## Results

### FGDs and Participants

In total, 3 FGDs were conducted with 15 patients (all females), 1 at each of the sites Arua, Mayuge, and Mbarara (n=5, 33.3%, in each FGD). In addition, 10 FGDs (5 at the start of the project, and again 5 after 4-6 months of operation) were conducted with 27 nurses and administrators at Arua (n=5, 18.5%), Jinja (n=5, 18.5%), Mayuge (n=6, 22.2%), Mbarara (n=5, 18.5%), and UCI Kampala (n=6, 22.2%). All the staff were female (nurses), except 3 (11.1%), who were male (administrators). Of the health workers, 20 (74.1%) completed the evaluation survey. Table 1 summarizes the characteristics of the participants.

**Table 1:** Participant distribution and characteristics.

Characteristics					Site								
			Arua	Jinja			Mayuge		Mbarara		UCI^a^ Kampala		Overall
**Patients**													
	Number, n (%)	5 (33.3)		0		5 (33.3)		5 (33.3)		0		15 (100.0)	
	Age (years), mean (SD)	29.2 (7.7)		N/A^b^		28.6 (2.7)		30.6 (6.9)		N/A		29.5 (5.8)	
**Health workers**													
	Number, n (%)	5 (18.5)		5 (18.5)		6 (22.2)		5 (18.5)		6 (22.2)		27 (100.0)	
	Age (years), mean (SD)	43.0 (5.9)		36.2 (5.8)		39.8 (10.1)		36.8 (8.5)		35.7 (4.8)		38.4 (7.7)	
**Health workers’ sex, n (%)**													
	Female	4 (16.7)		4 (16.7)		5 (20.8)		5 (20.8)		6 (25.0)		24 (88.9)	
	Male	1 (33.3)		1 (33.3)		1 (33.3)		0		0		3 (11.1)	

^a^UCI: Uganda Cancer Institute.

^b^N/A: not applicable.

### Perceptions of CaCx Screening Nurses Toward the Telemedicine System and AI

Generally, the CaCx screening nurses from all sites were positive about the intervention and considered it acceptable and useful. They mentioned the following as some of the perceived benefits or value additions of the system: (1) the ability to send an image for a second opinion when in doubt of the VIA results or for advice on the best treatment option for VIA-positive cases, depending on the size of the lesion; (2) the ability to consult and discuss with a colleague in the clinic using the image on the smartphone rather than keeping the patient long in the lithotomy position; (3) better illumination from the Gynocular unit’s light compared to ordinary torches; (4) better visualization of the cervix on the screen of the smartphone (with the possibility to zoom), both of which improve the nurses’ confidence and accuracy of assessment; and (5) ease of communicating findings to the patients by showing them images of their cervix. These benefits were highlighted both in the qualitative findings (Multimedia Appendix 6) as well as in the supplementary survey (Table 2), where participants also indicated that the project in general helped improve their interactions with patients and colleagues, research skills, and CaCx screening skills.

**Table 2:** Health workers’ (n=20) rating of the utility of the intervention and the usability of the system (based on the SUS^a^ [75]).

Question		Strongly disagree, n (%)	Disagree, n (%)	Neutral, n (%)	Agree, n (%)	Strongly agree, n (%)	Good usability (ie, agree+strongly agree), n (%)
**Please indicate the extent to which you agree or disagree with the following statements about the telemedicine system.**							
	I like using the telemedicine system.	1 (5.0)	0	1 (5.0)	10 (50.0)	8 (40.0)	18 (90.0)
	I would use the telemedicine system again.	1 (5.0)	0	1 (5.0)	8 (40.0)	10 (50.0)	18 (90.0)
	The telemedicine system is an acceptable way for carrying out CaCx^b^ screening.	1 (5.0)	0	1 (5.0)	5 (25.0)	13 (65.0)	18 (90.0)
	The app interface and navigation instructions are simple and easy to understand.	1 (5.0)	0	1 (5.0)	9 (45.0)	9 (45.0)	18 (90.0)
	Overall, I am satisfied with the telemedicine system.	1 (5.0)	0	2 (10.0)	10 (50.0)	7 (35.0)	17 (85.0)
	The telemedicine system for CaCx screening improves access to healthcare.	1 (5.0)	0	2 (10.0)	8 (40.0)	9 (45.0)	17 (85.0)
	I believe I could quickly become productive using this system	1 (5.0)	0	2 (10.0)	4 (20.0)	13 (65.0)	17 (85.0)
	The way I interact with patients using the telemedicine system is pleasant.	1 (5.0)	0	2 (10.0)	7 (35.0)	10 (50.0)	17 (85.0)
	I feel comfortable using the telemedicine system during CaCx screening.	1 (5.0)	0	2 (10.0)	7 (35.0)	10 (50.0)	17 (85.0)
	It was easy to learn how to use the technology or IT system in the project.	0	3 (15.0)	1 (5.0)	10 (50.0)	6 (30.0)	16 (80.0)
	It was easy to use the technology or IT system in the project.	0	2 (10.0)	4 (20.0)	8 (40.0)	6 (30.0)	14 (70.0)
	The telemedicine system saves time during CaCx screening.	0	2 (10.0)	5 (25.0)	9 (45.0)	4 (20.0)	13 (65.0)
	Whenever I made a mistake using the system, I could recover quickly and easily.	1 (5.0)	3 (15.0)	5 (25.0)	10 (50.0)	1 (5.0)	11 (55.0)
	The system gave error messages that clearly told me how to recover from the mistake.	2 (10.0)	3 (15.0)	6 (30.0)	7 (35.0)	2 (10.0)	9 (45.0)
**Please give your level of agreement with the following benefits from the project.**							
	Overall, I am satisfied with this project.	0	1 (5.0)	1 (5.0)	8 (40.0)	10 (50.0)	18 (90.0)
	The project helped me to improve my interaction with patients during screening.	1 (5.0)	0	2 (10.0)	4 (20.0)	13 (65.0)	17 (85.0)
	The project helped me to improve my research skills.	2 (10.0)	0	1 (5.0)	8 (40.0)	9 (45.0)	17 (85.0)
	The project helped me to improve my CaCx screening skills.	2 (10.0)	0	3 (15.0)	5 (25.0)	10 (50.0)	15 (75.0)
	The project helped me to improve my computer skills.	0	2 (10.0)	3 (15.0)	9 (45.0)	6 (30.0)	15 (75.0)
	The project improved my ability to collaborate and consult with colleagues during patient management.	2 (10.0)	0	3 (15.0)	8 (40.0)	7 (35.0)	15 (75.0)

^a^SUS: System Usability Scale.

^b^CaCx: cervical cancer.

In terms of usability, at least 16 (80%) of 20 nurses rated the system as having good usability (ie, reported “agree” or “strongly agree” on a 5-point Likert scale ranging from “strongly disagree” to “strongly agree”) on 10 of the 14 SUS items (see Table 2).

The enthusiasm and perceived value of the intervention was highest for nurses from remote sites and those who were new to CaCx screening. The nurses at urban centers (UCI Kampala and Mbarara) considered the intervention useful but not an absolute necessity, since they often have access to consultants in case they need a second opinion or because they felt they already know how to conduct VIA assessments. These nurses also pointed out that with busy clinics, their priority is on screening and treating all women, even if it means overtreating some, instead of spending precious time on getting a second opinion to ensure accurate VIA results. The concern that using the telemedicine system is time-consuming was echoed by other nurses at rural clinics and was also reflected in the usability survey, but in this case, it was in relation to the need for assembling the different components of the system (the smartphone, the Gynocular unit, harness, and stand). This concern was particularly reported at the start of the project, but with experience, this ceased to be reported.

Other challenges pointed out or observed included bad-quality images, especially at the start of the project (eg, due to positioning of the Gynocular unit too far or too close to the cervix); an occasional slow internet connection or depletion of broadband data bundles; and difficulties in setting up the Gynocular unit with the phone, which caused the Gynocular harness to obstruct the phone camera lens. The nurses were also concerned about the lack of technical support to repair the Gynocular unit when it breaks down.

When specifically asked about the AI, the nurses did not express any fear of AI threatening or replacing their role in the health care process. Instead, they were enthusiastic about the prospect of getting help from a machine when their skills are inadequate or when they are tired. They were particularly happy when there was agreement in the assessment between them and the AI, as this reassured them that they were managing patients correctly. Finally, in a small evaluation survey of the entire project, the AI was considered the most important component for patient care (n=8, 40%) compared to the other components (remote consultation: n=7, 35%; training of nurses on CaCx screening: n=2, 10%; creation of image data sets for AI research: n=2, 10%; and electronic clinical documentation: n=1, 5%).

### Perceptions of the Telemedicine System and AI by Administrators and Managers

Administrators and staff in charge of the clinics or in supervisory roles appreciated the intervention for providing access to *electronic data*, which allows them to obtain an overview of the screening activities and support research, including the development of AI. They also highlighted the value of the system in enabling reviews of the nurses’ VIA assessments by gynecologists and providing them with feedback. From the consultations as well as analysis of the assessments made by the nurses versus those by the gynecologist during the training of the AI model, the areas where some nurses were struggling (eg, identifying the squamocolumnar junction [SCJ] or differentiating between mucus plugs and aceto-white changes) were noted and feedback provided to them during site visits. Another perceived benefit was modernization of the screening service, which they thought would boost confidence in the clients and increase the uptake of screening services.

### Perceptions and Opinions About the Telemedicine System and AI by the Patients

Similar to the health workers, the patients were also generally positive about the intervention. They felt that it brought the consultant services closer to them, which otherwise would not be accessible. They also noted that with this technology, nurses would discuss among themselves, which showed them that they care. In addition, patients reported that nurses explained better using the images of the cervix. Even in cases where their condition would require a referral, patients felt that this intervention would ease the process since the referral site would already have their information. The nurses confirmed the patients’ perceptions and sentiments from their observations and interactions with them, saying that the women wanted to see their cervix and that they were happy knowing that consultants from a specialist hospital would be evaluating them.

We specifically explored the issue of privacy and confidentiality with patients, considering the sensitivity of the data. Generally, patients were not worried, due to their trust in the professionalism of health care providers, while others pointed out that since the images did not contain personally identifying information, there was no problem sharing them. A few were concerned but felt the benefit of getting screened and an accurate assessment outweighed the potential negative consequences. However, for 1 (6.7%) patient, this privacy concern caused her to refuse screening. Unfortunately, this happened before the nurses explained the purpose and operation of the system and how privacy concerns are addressed, so she only decided based on incomplete information she obtained from other patients. Otherwise, the nurses’ explanations and showing patients the captured images helped allay privacy fears.

### Observations and Experiences of the Implementation Team

In addition to the above-mentioned findings from the FGDs with health facility staff and patients, the research team made several observations that merit highlighting. A positive observation was that the time needed to train the nurses on the system (assembling the equipment set, capturing cervical images, and entering clinical data) was fairly short. It took only 2-3 half-day sessions spaced about 2 weeks apart with remote feedback and troubleshooting of issues that arose.

However, there were negative observations that could limit the impact of the intervention. First, there were few consultations or requests for a second opinion, which was the main aim of the intervention. These remote consultations were mostly at the start of the project and from the new sites (those operationalized during the project). Second, we faced difficulties in software development and integration with the existing EMR system UgandaEMR (a distribution of OpenMRS) [76]. Although at the UCI, there was no point-of-care EMR system at the time of project initiation, we intended to use the project to start the EMR adoption process. However, due to resource constraints of the project, and the high technical debt in customizing UgandaEMR to support cancer workflows, allow image capture for VIA, and incorporate the AI model, we decided to develop a custom web app. Third, there were a few patients with advanced lesions (suspicious for cancer) and needed referral, even after further assessment with teleconsultation. Lastly, there was no local technical support or service providers to repair a broken Gynocular unit, as well as a lack of sustainable funding as the project was running on grants.

## Discussion

### Principal Findings

In this paper, we presented the experiences of implementing a smartphone-based telemedicine system for VIA-based CaCx screening at 5 screening clinics in different regions of Uganda. Generally, the health workers, health facility administrators, and patients attending CaCx screening were positive about the intervention and perceived it as useful and acceptable with high usability. The main cited benefits were related to improved patient experience, particularly better communication and understanding of the screening process and results using cervical images, and improved referral processes. Other benefits included improved VIA through teleconsultation, the use of AI algorithms, and supervision and feedback; modernization of the screening process, thus boosting patient confidence and trust in the screening; and improved clinical documentation and data management to support AI development and research and monitoring.

This study is the first to report on the implementation of smartphone-based telemedicine and AI in CaCx screening in Uganda. Studies from Tanzania [34,40,42], Madagascar [39,43,49], Eswatini [44,45], Botswana [35,77], Ghana [36], and Kenya [55] have used smartphone-based cervicography and have reported similar results as ours, including the ease of learning and adoption of smartphone-based telemedicine setups by CaCx screening staff, good-quality captured images that allow correct assessment by remote experts, and enhancement of VIA accuracy and clinical data management by such digital tools. Other studies from India [38] and Costa Rica [46] have attempted to develop AI models for analysis of cervical images, although clinical deployment has not been fully realized. The reported benefits and acceptability by key stakeholders (health care workers and patients), infrastructural feasibility and technical simplicity of smartphone-based telemedicine, and enthusiasm in the AI and overall digitalization of CaCx screening were important facilitators for implementation of our intervention. They are also critical for long-term adoption [51-53,69-72]. In particular, problems with electricity were never reported in our study (since the Gynocular unit and smartphones use rechargeable batteries), and poor internet connectivity was also rarely a concern. This further demonstrates the potential for smartphone-based interventions to leapfrog these infrastructural limitations that are traditionally cited as barriers to technological adoption in LMICs.

An important contribution of this study to the CaCx control literature is that it provides insight into the role of digital health tools in improving patient experience of the screening process. This is an area that few prior studies have highlighted [36,40]. Improving the patient experience, for example, through a better explanation of the screening process and findings using cervical images, is important for improving patient satisfaction and trust in the screening, reducing fears and myths, and improving acceptance of the findings (eg, a positive VIA result even when asymptomatic). In turn, this increases the likelihood that women adhere to the treatment or referral, return for future screening, and encourage others in the community to take up screening through a snowball effect [78]. Given the low screening coverage, increasing uptake is critical for CaCx control in Uganda and other high-burden countries. Moreover, although new World Health Organization (WHO) guidelines [79] recommend using HPV screening as the preferred method, VIA will likely remain relevant in Uganda and other LMICs, given the logistical challenges that limit HPV screening, as well as the necessary transition period. Therefore, interventions to improve VIA [31], such as mechanisms for teleconsultation or the development of AI models, as described in this study, are necessary.

At the same time, negative perceptions and barriers must be addressed to ensure maximum impact and long-term adoption. In our study, barriers related to the technology used were minimal and surmountable. For example, the steep learning curve and perception that the system is time-consuming were reported only in the early phase of the project. Concerns about patient privacy and confidentiality were also easily addressed by proper explanation by the screening nurses. However, barriers related to the outer context beyond our intervention, such as attitudes and health system limitations, pose a bigger challenge.

### Limitations

The main objective of setting up this intervention was to facilitate remote consultation, but this only happened to a small extent. This could be explained by better visualization using the system and hence straightforward and confident assessment by the nurses at the front line. Indeed, when consultations were made, often these were about how to manage or coordinate referrals, rather than assessments. Another explanation for the limited consultations, especially at sites with more experienced nurses, is that these nurses seemed to not consider an inaccurate VIA assessment to be a problem. Instead, they were more concerned with screening as many women as possible, given the low screening coverage and high patient volumes. For them, screening is for triage where more accurate evaluation is performed by a gynecologist in case the VIA conclusion is not obvious. Nonetheless, these nurses saw value in other aspects of the intervention (eg, facilitating communication with patients and modernizing the screening process). This is an important issue to consider since adoption of digital interventions can be affected by misalignment of the users’ needs or pain points with the services of the digital solution or the problems it solves. Social marketing principles [80] can be used to understand the needs, aspirations, and preferences of the target users of digital health interventions, as well as aligning the sensitization, communication, and positioning of the intervention to these.

Another limitation of our project is the sustainability challenge. The project has been running on grant funding and has not been fully institutionalized. Although this has to do with the UCI’s nascent digitalization journey (or Uganda’s in general) characterized by fragmented pilots of different digital health systems and tools, it nonetheless undermines the long-term sustainability and impact of this project. Going forward, sustainability plans and efforts should be explored, including integration with existing digital health systems, as well as collaboration with other implementers of similar systems, so as to reuse any available (open source) artifacts. This could also save costs, for example, in collection and curation of AI training data sets by reusing data collected by others doing similar projects.

### Conclusion

This study shows that digital health tools are useful for improving the accuracy of VIA, as well as improving patient experience, which is crucial for CaCx screening uptake. However, general barriers to digital health in LMICs (eg, poor internet access and lack of interoperability), as well those specific for this telemedicine system (eg, the perceived extra burden on health workers in terms of time taken during screening using the system and concerns of patients about privacy), are also highlighted. These barriers must be addressed if telemedicine is to be harnessed to support CaCx control in Uganda.

## Appendix

Multimedia Appendix 1 The Gynocular unit and smartphone being used by nurses to screen for cervical cancer.

Multimedia Appendix 2 The ODK Aggregate server dashboard. ODK: Open Data Kit.

Multimedia Appendix 3 The data collection app with the AI model. Data are fictious. Notice the data validation features. AI: artificial intelligence.

Multimedia Appendix 4 Dashboard with AI results. Notice some disagreements between the AI (labeled “ML diagnosis”) and the nurse diagnosis. AI: artificial intelligence.

Multimedia Appendix 5 Focus group discussion guide.

Multimedia Appendix 6 Perceived benefits of the telemedicine system.

## Abbreviations

AI: artificial intelligence
CaCx: cervical cancer
CFIR: Consolidated Framework for Implementation Research
EMR: electronic medical record
FGD: focus group discussion
HPV: human papillomavirus
LMIC: low- and middle-income country
mHealth: mobile health
ODK: Open Data Kit
SUS: System Usability Scale
UCI: Uganda Cancer Institute
UNCST: Uganda National Council for Science and Technology
VIA: visual inspection with acetic acid

## Data Availability

The data sets generated and analyzed during this study are available from the corresponding author upon reasonable request.
