# Supplementary figures and images for: Implementing Smartphone-Based Telemedicine for Cervical Cancer Screening in Uganda: Qualitative Study of Stakeholders’ Perceptions
Source: J Med Internet Res. 2023 Oct 2;25:e45132. doi: 10.2196/45132 (PMC10580134; doi:10.2196/45132)

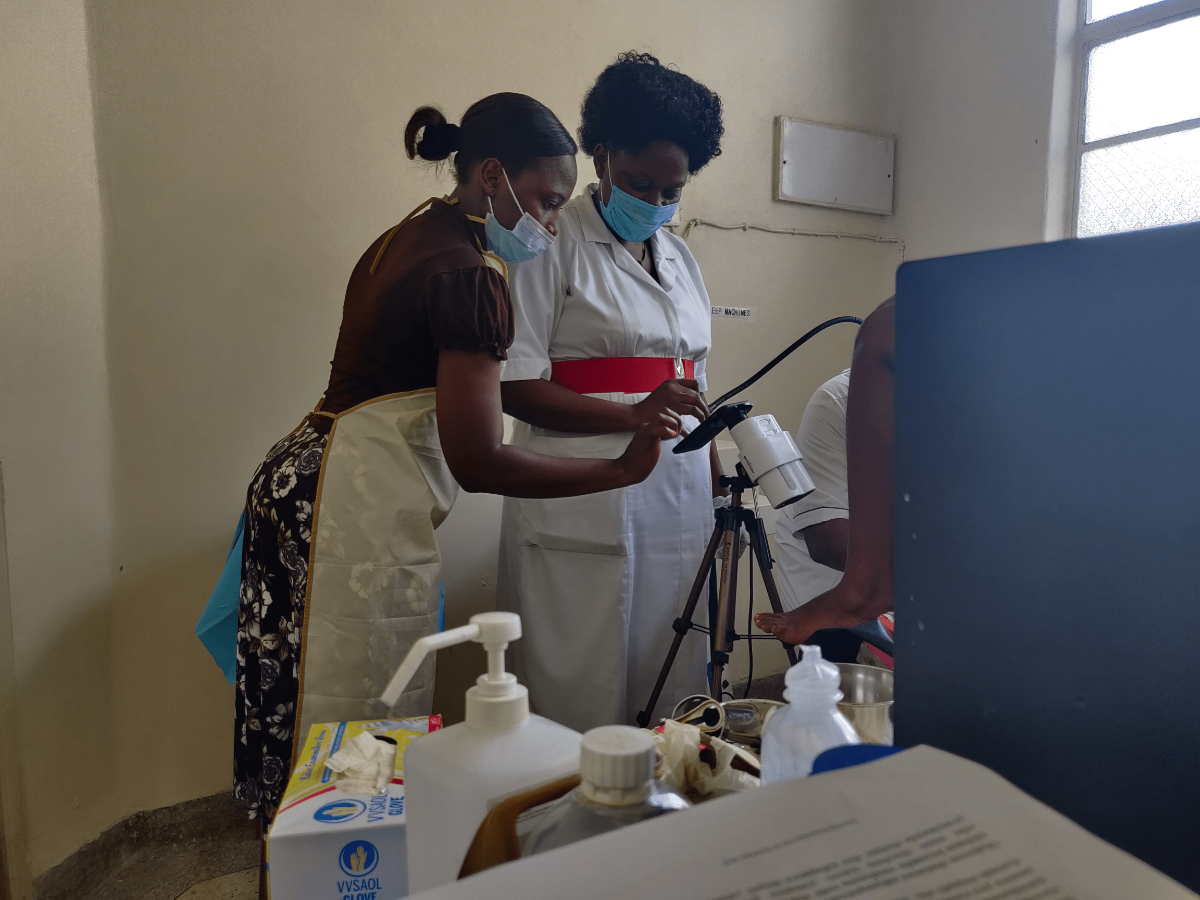

Supplement: Multimedia Appendix 1 [file jmir_v25i1e45132_app1.png]

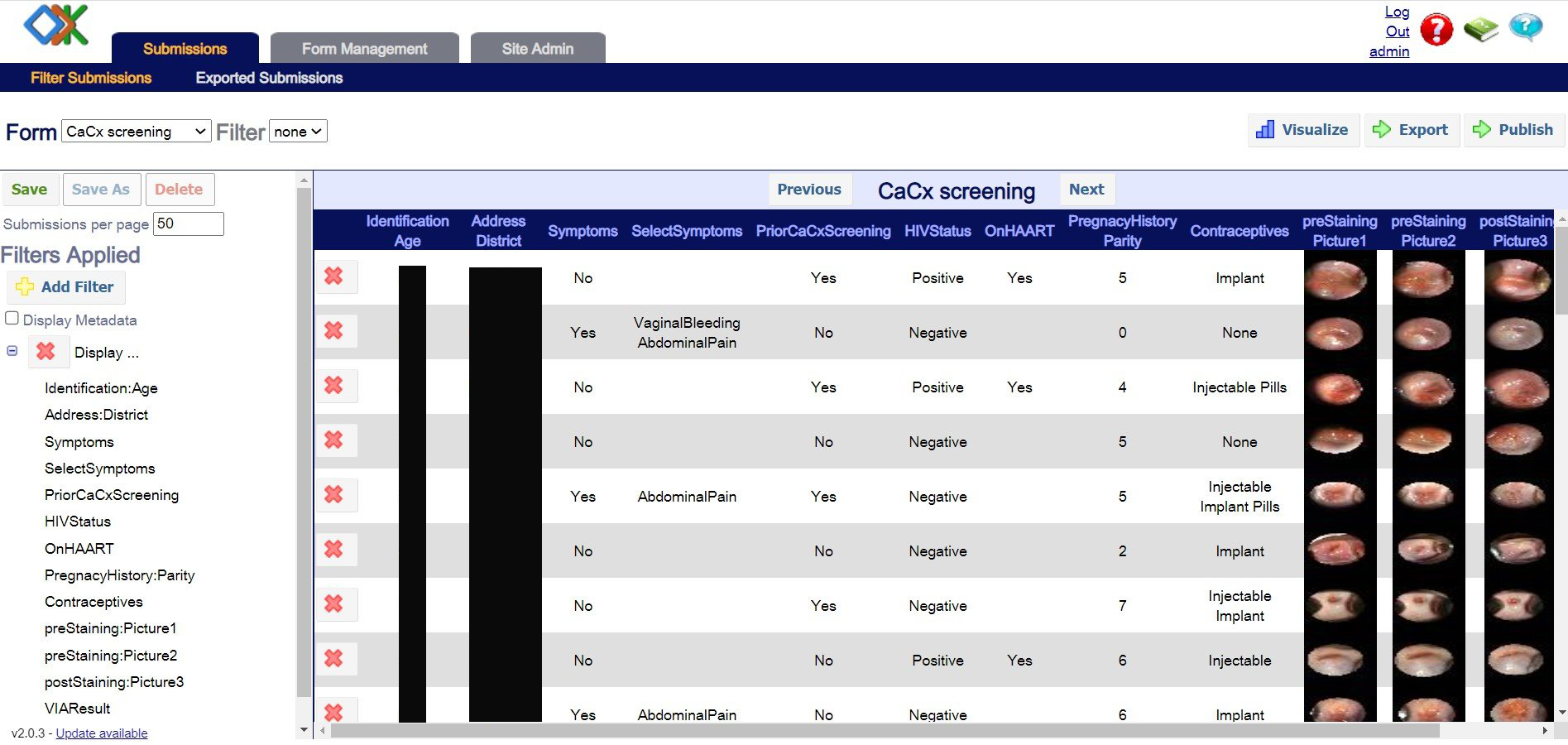

Supplement: Multimedia Appendix 2 [file jmir_v25i1e45132_app2.png]

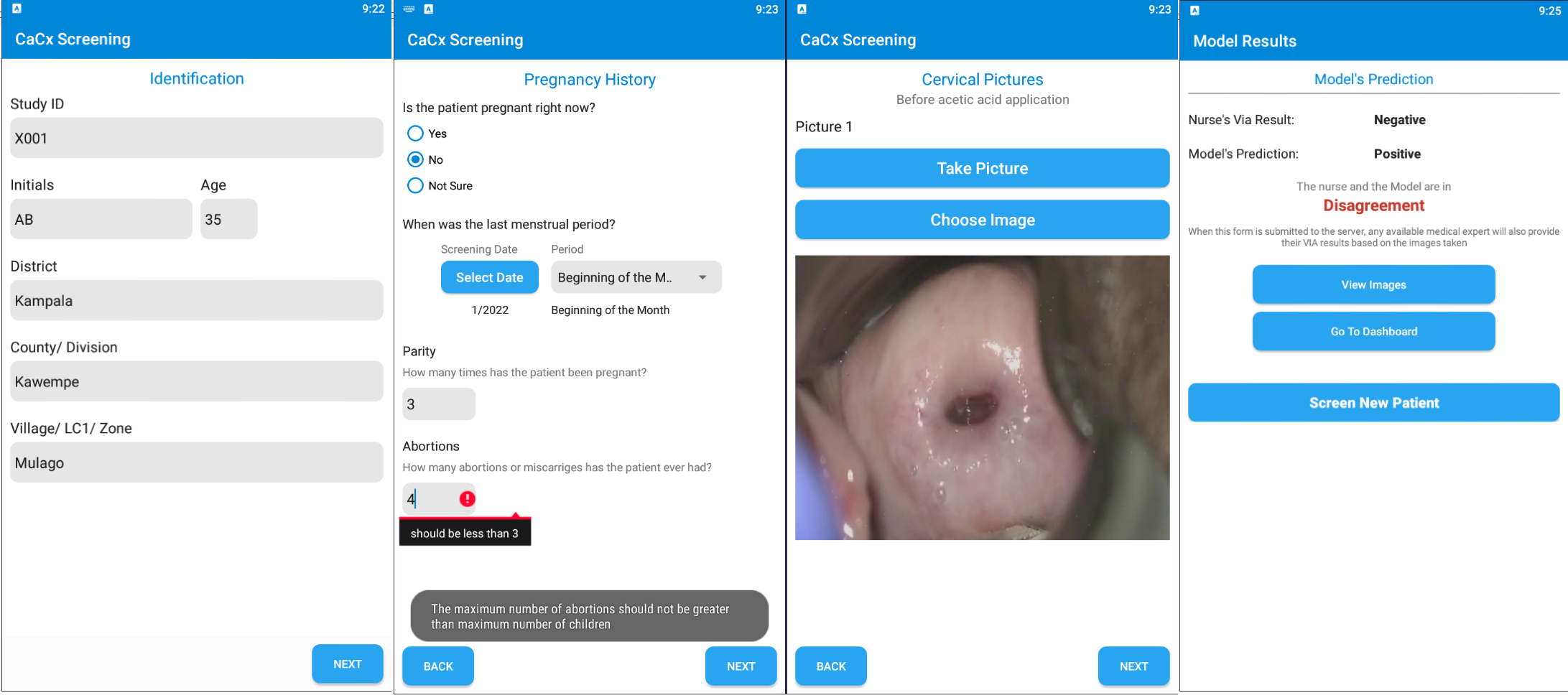

Supplement: Multimedia Appendix 3 [file jmir_v25i1e45132_app3.png]

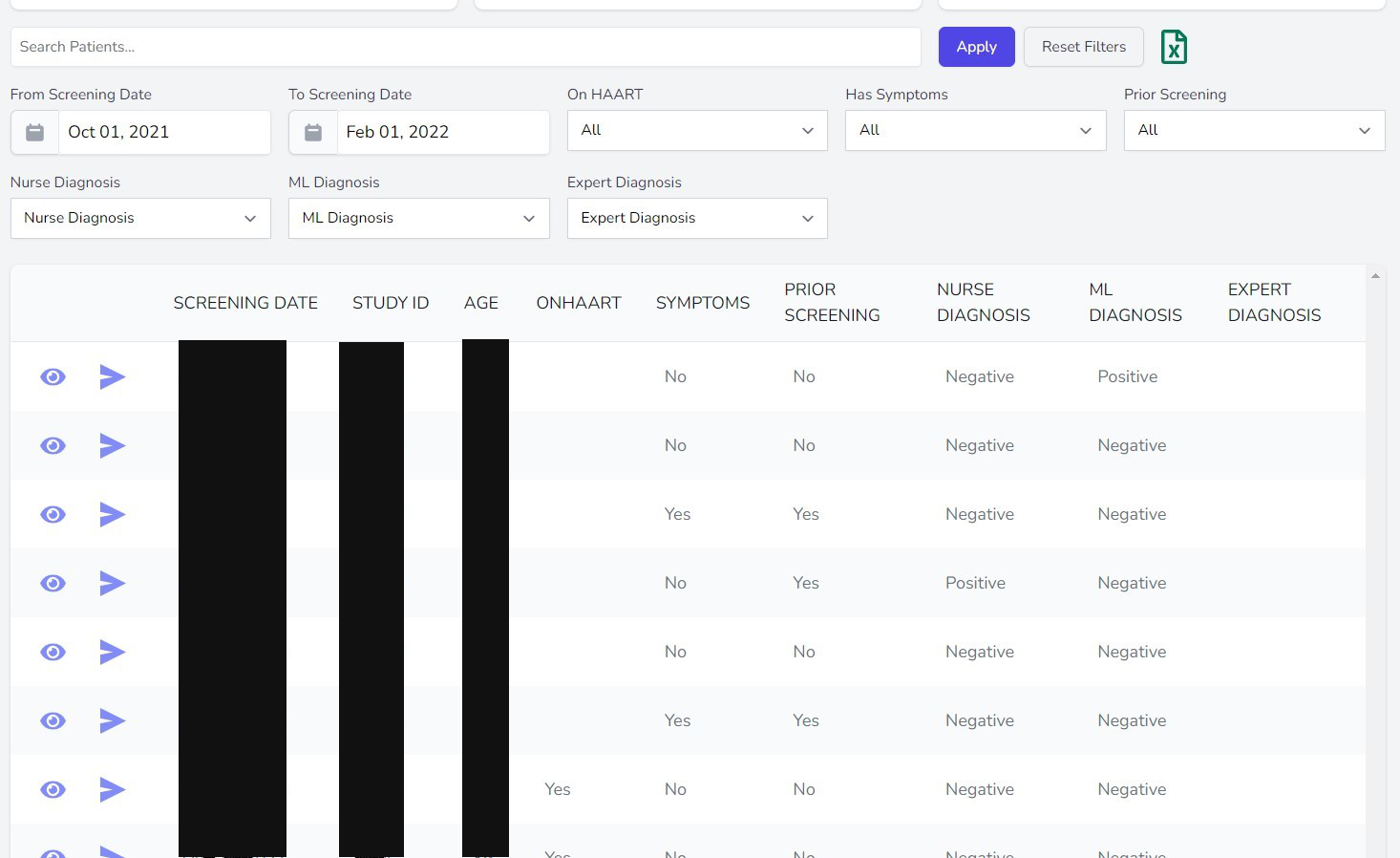

Supplement: Multimedia Appendix 4 [file jmir_v25i1e45132_app4.png]
