# Supplementary material for: Implementing Smartphone-Based Telemedicine for Cervical Cancer Screening in Uganda: Qualitative Study of Stakeholders’ Perceptions
Source: J Med Internet Res. 2023 Oct 2;25:e45132. doi: 10.2196/45132 (PMC10580134; doi:10.2196/45132)
Supplement: Multimedia Appendix 5 [file jmir_v25i1e45132_app5.docx]

**FOCUS GROUP DISCUSSION GUIDE**

Experienced moderators will lead the discussions and will use probes to explore the following issues:

- Patients’ opinions and experience of mobile colposcopy, their fears or concerns, perceived benefits and barriers (e.g. on privacy and sharing information electronically, second opinion about diagnosis from a remote health worker, time saving, transportation cost saving), satisfaction with care, etc.
- Health workers’ experience and opinion of mobile colposcopy: learning or knowledge sharing (do they learn from their colleagues at the remote site to improve their VIA skills? Are there fears about their knowledge and skills being questioned or judged by the other colleagues at the remote site?), convenience and fit into workflow (time efficiency, technology skills demands, patient interaction - does technology get into the way of patient care?), satisfaction with care provision
- Health workers’ experience and opinion of using the machine learning model at the Point-of-Care: Are there fears about their knowledge and skills being questioned or judged by the other colleagues at the remote site?), convenience and fit into workflow (time efficiency, technology skills demands, patient interaction - does technology get into the way of patient care?), satisfaction with care provision, usability of the AI application, key technical issues with ethical implications of using the AI model.
- Observation of time efficiency: The clinicians who have used the Gynocular will be asked to subjectively describe their experience with respect to time taken at different stages of cervical cancer screening e.g. from when a patient enters the clinic to when they are done with the screening, time for documentation, time for getting feedback or opinion of the gynaecologist by the nurse in cases where the impression is not clear or consultation is needed, etc.
- Explore men’s opinions about mobile colposcopy: What do they, as husbands of women who go for cervical cancer screening, think of telehealth solution (mobile colposcopy) in screening with respect to privacy, patient experience, etc?
