# Supplementary material for: Implementing Smartphone-Based Telemedicine for Cervical Cancer Screening in Uganda: Qualitative Study of Stakeholders’ Perceptions
Source: J Med Internet Res. 2023 Oct 2;25:e45132. doi: 10.2196/45132 (PMC10580134; doi:10.2196/45132)
Supplement: Multimedia Appendix 6 [file jmir_v25i1e45132_app6.docx]

Summary of qualitative findings showing themes (based on constructs from the CFIR^a^ [69-72]), along with illustrative quotes and classification into barriers or facilitators.

| Theoretical construct from the CFIR and finding | | Illustrative quotes | Implication for implementation or adoption |
| --- | --- | --- | --- |
| **Relative advantages (or perceived benefits)** | | | |
|  | The telemedicine system offers several benefits compared to the traditional way of screening. These include (1) the ability to send an image for a second opinion or advice on treatment options, (2) the ability to consult and discuss with a colleague within the clinic, (3) better illumination from the Gynocular unit’s light, (4) better visualization of the cervix, and (5) ease of communicating findings to the patients. | - “With my colleagues, it helps us consult each other. If you are not sure, you call a colleague and you look at the picture together and conclude. We also call the UCI^b^ for a second opinion. Before, if I would need to ask a colleague, I would remove the speculum, find the colleague, and reinsert the speculum to show her. But now, I show her the picture.” [Nurse at Mayuge] - “…we have found that compared to using a plain eye, the Gynocular gives a better picture…magnification allows better visualization…we are more confident in the assessment we make.” [Nurse at UCI Kampala] - “The patients are more appreciative when you show them the picture of their cervix. You show them the picture before and after applying acetic acid. So, when you are explaining to them the changes, they appreciate it.” [Nurse at UCI Kampala] - “With this, if you are referred the process will be easier. The people at Mulago will already have your information.” [Patient at Mayuge] - “Compared to my experience the last time I screened, this time it was good. They explained to me better.” [Patient at Mayuge] | Facilitator |
| **Recipient centeredness (culture)** | | | |
|  | The patients also appreciated the improved communication and the fact that health workers were consulting and discussing amongst themselves, which meant that they cared about accurately assessing patients. | - “The difference I am seeing is that the other time there was only one person who was attending to me and today there were many people discussing.” [Patient from Arua comparing the previous time she went for CaCx screening] - “Compared to my experience the last time I screened, this time it was good. They explained to me better.” [Patient at Mayuge] - “The patients are more appreciative when you show them the picture of their cervix. You show them the picture before and after applying acetic acid. So, when you are explaining to them the changes, they appreciate it.” [Nurse at UCI Kampala] | Facilitator |
| **External pressure (market pressure)** | | | |
|  | The health workers also felt there was a need to modernize the screening process, and the system contributed to this modernization, which increases trust and uptake by patients. Patients also stated that they would encourage others to come for screening now that there was a machine, while others were heard doing so by the research team. | - “I even feel like the service we have here is now modernized. Because of the technology [more patients] will come; it gives them that hope of coming. The moment this message will go out there that there’s now an improved screening method in the hospital, you will see a number of them.” [Administrator (gynecologist) at Arua] - “Traditionally, people think that when you go for a checkup for a disease like cervical cancer, there are a lot of machines involved. So, if you do a procedure with just acetic acid, somebody does not feel satisfied. So, now with this technology, we are going to see more people coming for screening. Previously, people were reluctant, but now, they think, ‘They have brought machines from Mulago.’ They are now confident, not just the simple checking [with naked eye].” [Administrator at Mayuge] - “Since we have been tested today by the machine and are sure of safety of our test results, we shall tell our friends not to be worried, since we’ve acted as an example.” [Patient at Mbarara] | Facilitator |
| **Innovation complexity and design** | | | |
|  | The system was time-consuming, particularly at the start of the project when the system had just been introduced. This was mostly attributed to the need to assemble the different components (smartphone, Gynocular unit, and the harness and stand), but some nurses also had difficulty at first in taking good-quality images (ie, the right distance from the cervix and focusing with the camera). | - “Yes it is good, but it is time-consuming.” [Nurse at UCI Kampala] - “For me, at first, it was really disturbing to focus and get a clear picture, and I think those other people from a satellite clinic are likely to face the same [challenge], but as you get experience, you get a clear picture.” [Nurse at UCI Kampala] - “The time has increased from the other time we were screening just by VIA and the client goes. Since here, you screen by VIA still, but you have to take a photo…it has increased by like 3 minutes” [Nurse at Mayuge] - “I wish all the equipment were assembled for use…put together as a single gadget.” [Nurse at Mayuge] | Barrier |
| **Local conditions/IT infrastructure** | | | |
|  | Slow internet connectivity occasionally affected the system. | - “Internet…it’s about internet because we may want a quick reply [second opinion], but when the photo has refused to go in real time, it goes at the end of the day, when the patient has already left. That is inconveniencing the patient…we tell them it has failed to go, but maybe you come back tomorrow…we always get their telephone numbers [to send the diagnosis later].” [Nurse at Mayuge] | Barrier |
| **Critical incidents** | | | |
|  | The nurses were concerned about the lack of technical support for when system components break down or malfunction. The research team also observed a portable colposcope that was out of use after breaking down. | “…we need technicians closer to us, because anything small, you’re not technical, you can’t touch, so as we are doing this, think about the technicality if we have a problem, who can come first to help us solve the problem because machines can come and within a short time they are down, they cannot work, and we don’t have people to come and do the repair.” [Nurse at Mbarara] | Barrier |
| **Attitudes** | | | |
|  | There was no threat to professional autonomy or job security, but rather, nurses felt AI^c^ had a separate and collaborative role, that a second opinion was sometimes (including when they are tired) needed, and the system facilitated this. | - [Interviewer: So, you don’t mind about losing your jobs?]   “(Laughter). No because it is us that the machine will be telling so we shall not lose our jobs.” [Nurse at Arua]   - “It is a machine…it should not get tired. You know my eyes may get tired, but I don’t expect the machine to get tired.” [Nurse at Mbarara] - “I think it will even bring more patients for us to screen after one being screened, then she goes out to tell [others] that now things are like this.” [Nurse in charge at Mbarara’ - “Patients are more confident knowing that someone else will give a second opinion.” [Nurse at UCI Kampala] - “Many mothers are coming here and [asking], Musawo, I want to be screened with the photo. It has helped, more clients are coming. They say, I want to see my cervix” [Nurse at Mayuge] | Facilitator |
|  | Accuracy of screening was not considered important in screening by some nurses, and therefore, the system was not considered an absolute necessity for its role in facilitating a second opinion. This was particularly so for nurses in busy clinics or where there are gynecologists to quickly provide a second opinion. | - “Here we are serving masses. And with VIA, you can really see with your naked eye, except in a few cases where there is a lesion and you want a second opinion on how to treat. From what I have observed, it does not add much more to what we are already doing. Because for screen and treat, the clients are not really sick, so when they come and you screen them, if you send them home, they might never come back, so if there is a lesion, you treat it. Besides, when I want a second opinion, here I can easily get it. Maybe in the rural setting. Here it can be there, but it is not a must to seek a second opinion all the time.” [Nurse at UCI Kampala] | Barrier |
| **Acceptability** | | | |
|  | For some patients, particularly if the protection measures in the system are not explained and they are not reassured of their privacy, the threat to privacy (the fact that pictures of their cervix were being captured and shared) caused them to refuse to undergo screening. | - “One client asked me, ‘That phone, are you going to share the pictures on Facebook?’ So, I had to explain that all apps were removed and we send only to [the] UCI. At first, they thought we were taking pictures of the whole body, but we explained we were just taking the cervix. And after taking the photo, we show them.” [Nurse at Mayuge] - “I was a bit uneasy, wondering where the images were being sent, but because we are interested in the screening and being healthy, we accepted. But there was one woman who left when she heard about images being taken. But after going through the procedure, I am no longer worried.” [Patient at Mayuge] | Barrier |
|  | Despite some concerns about privacy, other patients considered the benefits outweighed the risks. Moreover, they were comforted by the fact that images did not contain their identification information and that the health workers are professionals who would ensure the patient’s privacy is protected. | - “I don’t fear, since the picture does not contain my name on it, so even if the doctor spreads it on the internet, I won’t be worried. And this is a hospital; hospitals employ educated people.” [Patient at Mbarara] - “Since I was interested in the results, it wasn’t a problem to me; if it was for no reason, I would have declined, but since it was for screening, then I found no problem with it.” [Patient at Arua] - “It’s okay so that the people in Kampala are also able to see and know what the problem is; if this one misses something, the other one will be able to see, and this will help more women from this side” [Patient at Mbarara] | Facilitator |

^a^CFIR: Consolidated Framework for Implementation Research.

^b^UCI: Uganda Cancer Institute.
